# Comparison of the Penetration Depth of Conventional and Nano-Particle Calcium Hydroxide into Dentinal Tubules

**DOI:** 10.22037/iej.v12i3.16421

**Published:** 2017

**Authors:** Vahid Zand, Hadi Mokhtari, Aila Hasani, Golchin Jabbari

**Affiliations:** a *Department of Endodontics, Dental School, Tabriz University of Medical Sciences, Tabriz, Iran; *; b *Department of Endodontics, Dental and Periodontal Research Center, Dental School, Tabriz University of Medical Sciences, Tabriz, Iran*

**Keywords:** Calcium Hydroxide, Dentinal Tubules, Nano Particle, Penetration Depth, Tubular Penetration

## Abstract

**Introduction:**

The aim of this *in vitro* study was to evaluate and compare the penetration depth of conventional (CH) and nano-particle calcium hydroxide (NCH) into dentinal tubules.

**Methods and Materials:**

Ninety human single-rooted teeth were instrumented by RaCe rotary system and after chemomechanical preparation were randomly divided in two equal groups (*n*=45). In the first group conventional CH and in the other NCH was used as intracanal medicament. After 2 weeks of incubation all roots were intentionally split at longitudinal axis and prepared for scanning electron microscope (SEM) observation. Three zones of each root, coronal, middle and apical were examined under SEM and the maximum penetration depth of the dressing material into dentinal tubules was recorded for each zone. Data were analyzed using the independent sample *t *test and the level of significance was set at 0.05.

**Results:**

In all of the three zones, NCH group had greater penetration depth than CH (*P*<0.001). In both groups the penetration depth increased from the apical section to the coronal.

**Conclusion:**

The depth of penetration of nano-particle calcium hydroxide into the dentinal tubules was significantly higher than that of conventional calcium hydroxide. The lowest penetration depth was observed in apical zone in both groups.

## Introduction

The chief aim of root canal therapy is to eliminate microorganisms from the root canal system and prevent reinfection to achieve complete periradicular healing [1]. Even after thorough mechanical instrumentation and irrigation, due to complexities of the root canal system, bacteria may not be completely eliminated [2]. Therefore, an antimicrobial intracanal medicament is needed to enhance the success rate. The material of choice for intracanal medication is calcium hydroxide (CH) [1, 3, 4]. It has many beneficial properties, such as antimicrobial activity [5], potential to induce hard tissue formation, capacity to limit inflammation and substitutive root resorption [6] and dissolution of organic tissues [7]. In order to bring about a favorable effect, CH should penetrate into dentinal tubules to come into direct contact with microorganisms.

Nano-particles are microscopic particles, measuring less than 100 nm in size [8].Various nano-particles have become popular in dentistry and medicine as antimicrobial agents [9]. The higher surface-to-volume ratio and charge density result in their greater interaction with the environment, and leads to their higher antibacterial activity [10-12].

An *in vitro* study showed that antimicrobial activity of nano-calcium hydroxide (NCH) was superior to conventional calcium hydroxide in culture medium [13]. In a study by Komabayashi *et al. *[14] on the size and shape of CH particles, they found that as the particle length decreased, the particle shape became rounder and as the particle lengthened, the particle shape changed to a more rectangular shape. So, it seems, short particles are more desirable for deep penetration into the dentin. Thus minimizing the particle size and production of nano form of CH may develop the penetration of the material into the dentinal tubules and also may enhance the antimicrobial efficacy due to the longer time of drug's presence in dentinal tubules. So the aim of this study was to evaluate and compare the penetrability of conventional and NCH into radicular dentinal tubules by using a scanning electron microscope (SEM).

## Materials and Methods

***Preparation of CH nanoparticles***

Preparation of NCH particles was done by peptisation with 2-propanol. By using Ca (NO_3_)_2_._4_H_2_O as the precursor, ethane-1,2-diol (ED) as the medium and aqueous NaOH as the precipitant, the hydrolysis method was applied. The procedure was adapted from Roy and Bhattacharya [15].

***Sample selection and specimen preparation***

Ninety recently extracted human single-rooted teeth were selected for this study (inclusion criteria: closed apices, no caries on the root surface, no external/internal resorption and no canal calcification). An ultrasonic scaler was used to clean up calculus and soft tissues. Then they were stored in 0.5 % chloramine-T solution until used for the purpose of the study. The tooth crowns were cut at cementoenamel junction using a #2 diamond disk (Tizkavan, Tehran, Iran) to have an easy access to canals. A #15 K-file was inserted into the canal until just become visible through the apical foramen and then 1 mm was subtracted from the measured length to obtain working length. After working length establishment the exterior of the apex was covered by utility wax. The instrumentation was performed by using crown-down technique with RaCe rotary system (FKG Dentaire, La-Chaux-de Fonds, Switzerland). The canals were instrumented up to 30/0.06 to working length. A #30 master apical file was considered for all the samples. Each root canal was irrigated with 2mL of 2.5 % NaOCl solution during instrumentation using an irrigation syringe with a 30-gauge needle inserted into the root canal 2mm short of the working length. To remove smear layer the final irrigation was carried out with 5 mL of 17% EDTA for 3 min, 2 mL of 2.5 % NaOCl for 3 min and finally with normal saline solution. The samples were randomly divided into two groups, each composed of 45 teeth. In one of the groups CH used as intracanal medicament and in other group NCH was used. To prepare the intracanal medicament, 100 mg of CH powder (Merck, Darmstsdt, Germany) or NCH was dissolved in 0.5 mL of normal saline solution. Then normal saline solution was added slowly until its final volume was 1 mL and a doughy consistency was achieved (25), which was carried into the prepared root canals with the use of a lentulo spiral and #40 paper cone (Ariadent, Tehran, Iran). Finally the coronal access was covered with utility wax and the teeth were incubated at 37^°^C and 100% relative humidity for 14 days. All the experimental procedures were performed by same operator.

***Sample evaluation by using a SEM***

**Figure 1.**
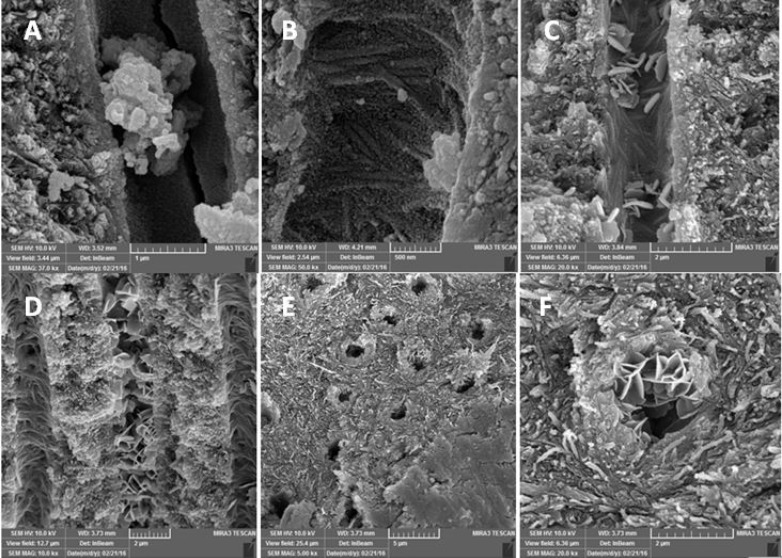
Scanning electron microscope images of experimental groups: A and B) Nano-particle calcium hydroxide in dentinal tubules; C and D) Conventional calcium hydroxide in dentinal tubules; E and F) Conventional calcium hydroxide particles stuck at the orifice of dentinal tubules, because of their rectangular shape

In order to evaluate penetration of CH into dentinal tubules, longitudinal sections were prepared. On opposite external surfaces of each root; two parallel longitudinal grooves were made by the means of a diamond disk, without penetrating into the canal space. Then the roots were split using end-cutting pliers. And the maximum depth of penetration for CH and NCH particles into the dentinal tubules was measured (µm) under a MIRA3 FEG-SEM (Tescan, Brno, Czech) at coronal, middle and apical thirds of each sample (Figure 1).To rule out any discrepancy a single operator assessed all the specimens. The data were averaged to have a single value per section.

***Data analysis***

Statistical analysis was performed using one-way ANOVA test for comparisons among 3 subgroups of each group. The post-hoc comparisons by the Tukey’s test were used to determine whether the differences between subgroups were statistically significant, or not. To compare the penetration depth of the materials in same sections of two groups, independent sample *t *test was carried out. Statistical Package for Social Science (SPSS, version 18.0, SPSS, Chicago, IL, USA) was used and the level of significance was set at 0.05. 

## Results

Table 1 shows the mean±SD of the penetration depth (µm) for the CH and NCH at three different zones. The ANOVA analysis results showed significant differences in penetration depth at three sections, in both groups (*P*<0.001). The Tukey’s test showed, significantly lower penetration depth of CH in the apical area than in the middle/coronal area (*P*<0.001). But the difference between coronal and middle section was not significant (*P*=0.588). However in NCH group the penetration depth increased from apical to coronal (*P*<0.001). Pairwise comparison between same sections of the study groups, using independent *t *test, showed that all sections in NCH groups had greater values for penetration depth than same sections of CH group and the difference was statistically significant (*P*<0.001).

**Table1:** Mean (SD) of penetration depth (µm) of CH and NCH

	**Coronal**	**Middle**	**Apical**
**CH**	125.6 (64.1)	115.2 (51.4)	28.3 (27.7)
**NCH**	454.7 (284.5)	303.6 (199.2)	110.5 (79.3)

## Discussion

Several studies have evaluated the penetration depth of the materials into dentinal tubules by the means of SEM [16-18]. Most important advantages of this technique are producing highly detailed images of dentinal tubules and their content and allowing observing the material within the tubules at distant region from the root canal wall. The main disadvantage is the difficulty of making systemic analysis at low magnifications. The potential for producing artifacts during the preparation is the other flaw.

The smear layer removal process exposes the orifices of dentinal tubules so increases the materials penetration into the tubules, although to varying depths [17, 19]. Therefore in this study the smear layer was removed by using of NaOCl and EDTA in both groups to allow better penetration of the medicament. 

**Figure 2:**
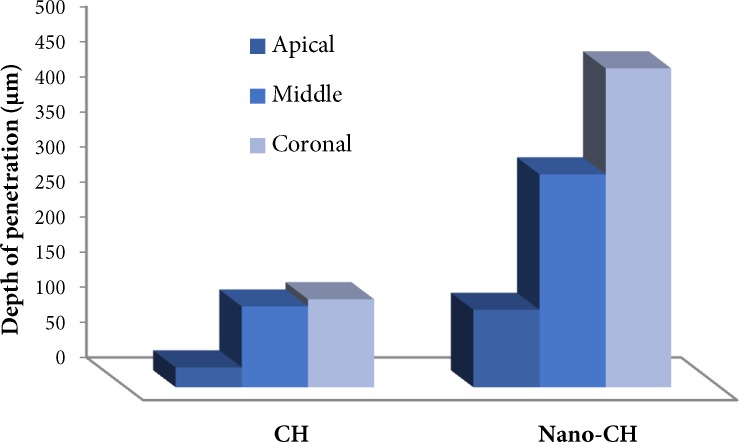
Zone-wise penetration depth of conventional and nano calcium hydroxide

CH has many advantages as an intracanal dressing. It has antimicrobial capacity and ability to hydrolyze LPS of gram negative bacteria [5]. Its high pH value promotes healing, calcification and hard tissue formation [20, 21]. Its diffusion through dentinal tubules may halter external root resorption and accelerates healing process [21-23]. Although, not as good as NaOCl, it has the ability to dissolve tissue debris [24].

Despite the antimicrobial activity of CH, it does not have a desirable effect on *E. faecalis *[5, 25-27], the bacteria which is the most prevalent microorganism involved in endodontic failures [28, 29]. One of the reasons may be the deep penetration of this bacteria into dentinal tubules (300 µm) and also the buffering capacity of dentin which preserves the bacteria from CH effect [30]. *E. faecalis* has been reported to withstand the high pH levels due to its acidifying cytoplasm and a functioning proton pump which helps the microorganism to maintain pH homeostasis. This resistance to high pH levels is the other reason for the shortcoming of CH to eliminate the bacteria. However *E. faecalis* does not survive at pH 11.5 or greater [31]. Dianat *et al. *[13] reported than NCH had superior antimicrobial activity against

*E. faecalis* compared to conventional CH in culture media, and also in dentin block models, NCH was more effective in 200 and 400 µm depths.

Komabayashi *et al. *[14], reported that size, shape and direction of calcium particles may control the penetration depth into dentinal tubules. As the particles increased in size, the particle shape became more rectangular but as the size decreased, the particles shape became rounder and therefore became more desirable for deep penetration.

In Figures 1E and 1F conventional CH particles are shown stuck at the orifice of dentinal tubules, because of their rectangular shape. The result of our study showed that NCH particles had more penetration depth compared to CH which is in consistent with what Komabayashi *et al. *[14] reported.

When the CH particles penetrate into the tubules they will be in direct contact with microorganisms, and also they may act as a direct source of dissociated CH which could continuously dissolve into aqueous form and produce OH^⁻^ ions so the pH will remain at high levels. Therefore antibacterial activity will be more strong and effective and there would be a slight chance of pH reduction by dentin buffering.

In this study penetration depth of both CH and NCH reduced from coronal to apical, and the least penetration depth was in apical portion (Figure 2). Previous studies had shown that tubular density decreases toward the apex and so does its penetrability. Apical dentin is more frequently sclerosed and contains areas without tubules [32]. The effectiveness of smear layer removal reduces in apical third of the root [33]. All of these explain why the penetration depth decreases toward the apex.

It is suggested to evaluate pH levels of CH and NCH at different depths of dentinal tubules, to assess how dentin buffering could have an effect on alkalinity of the materials. In addition to intracanal medication, other clinical usages of NCH, especially in regenerative treatments should be assessed in future studies.

## Conclusion

Under the parameters of the present study nano-calcium hydroxide particles exhibited deeper penetration into dentinal tubules in comparison with conventional calcium hydroxide. Thus they may act as a direct source of dissociated calcium hydroxide to continuously dissolve and produce OH^⁻^ ions and high pH levels. Penetration depth of both conventional and nano-calcium hydroxide decreased from coronal to apical, so minimum penetration depth was seen in apical portion for both groups.
